# Atypical Neuropsychiatric Presentation of FTD-ALS Caused by a Pathogenic Repeat Expansion in *C9orf72*: A Case Report

**DOI:** 10.1177/08919887231195337

**Published:** 2023-08-07

**Authors:** Marissa A. LeBlanc, Amy Gough, Andrea L. Rideout, Sarah Dyack, Kathleen Singh, Meagan MacNeil

**Affiliations:** 1 Department of Psychiatry, 3688 Dalhousie University, Halifax, NS, Canada; 2 432234 Nova Scotia Health, Halifax, NS, Canada; 3 IWK Maritime Medical Genetics, Halifax, NS, Canada

**Keywords:** c9orf72, frontotemporal dementia, psychosis

## Abstract

The case report describes the presentation of a 42-year-old male ultimately diagnosed with FTD-ALS caused by a genetic mutation, who initially presented with atypical psychiatric symptoms. Given that the initial clinical manifestations of FTD-ALS can be quite variable, the diagnosis is often challenging; the case report aims to highlight several key considerations in the diagnostic assessment, including genetic testing in order to guide clinicians in more timely diagnosis and ultimately improve patient care.

## Introduction

Frontotemporal dementia (FTD) and amyotrophic lateral sclerosis (ALS) are fatal neurodegenerative diseases that cause progressive morbidity for patients and personal burden for their families. As with other neurodegenerative diseases, the underlying etiology remains poorly understood, and there is no disease-modifying treatment. However, within the last decade, the hexanucleotide GGGGCC repeat expansion in the *C9orf72* gene was identified as the most frequent cause of both ALS and FTD in Europe and North America,^1,2^ with up to 10% of cases identified in the combined presentation of FTD-ALS.^3,4^ *C9orf72* related FTD-ALS is an autosomal dominant disorder, with a 50% recurrence risk in first degree relatives. Although the specific molecular mechanisms leading to neurodegeneration in patients with pathogenic expansions in C9*orf*72 have not been fully elucidated, there is evidence that these lead to alteration of various metabolic pathways in affected cells.^5^ The discovery of *C9orf72* gene expansion’s probable role in the underpinning of neurodegenerative disorders has contributed to the understanding of the inheritance of these disorders.

The clinical manifestations of neurodegenerative illnesses due to *C9orf72* repeat expansions are quite heterogenic with overlapping features.^6^ The most common clinical presentations are behavioural variants of FTD (bvFTD), ALS, or a combination of both (FTD-ALS).^7^ Neuropsychiatric symptoms due to *C9orf72* mutations most often include apathy, disinhibition, loss of empathy, stereotyped/repetitive behaviour, and hyperorality.^8^ However, late-onset psychotic disorders have been identified in some cases as the initial presentation of both FTD and ALS symptoms,^9,10^ with an increase prevalence of psychosis in individuals with *C9orf72* mutations.^11^ Other reports include a constellation of psychiatric presentations, including mania and bipolar disorder, major depressive episodes with and without catatonic features, and obsessive-compulsive disorder.

The wide variability in presentations associated with *C9orf72* pathogenic variants can make clinical diagnosis challenging. Improving the awareness of clinical presentations and when to consider genetic testing will help provide more timely diagnosis and ultimately improve patient care. In addition, attention to gene content is required when arranging genetic testing for FTD-ALS as testing for *C9orf72* expansions may need to be specifically requested in certain institutions. We present a case of FTD-ALS due to a pathogenic GGGGCC repeat expansion in *C9orf72* that first came to clinical attention with atypical psychiatric symptoms.

## Case Report

A 42-year-old previously healthy male with a remote history of mild depression presented to the local emergency department (ED) accompanied by concerned colleagues due to reports of personality changes and bizarre behaviour over the past several months. The initial unusual behaviours included being more intrusive, verbally aggressive, fixated on his mother’s health, and somewhat disinhibited with emotional lability (eg showing pictures of his deceased father to strangers and expressing excessive grief despite him dying more than 10 years prior). Due to these concerns, he had recently been compassionately released from his professional line of work. In his personal life, he was noted to be more erratic – eg dropping his children off and not returning to pick them up.

Upon initial assessment in the ED, there were no acute medical concerns identified, and psychiatry was consulted for assessment. Additional history obtained included a 1-year history of gradually progressive behavioural changes – eg, aversion to meat/proteins, with simple carbohydrate cravings; increased alcohol consumption; confrontations with the police; disinhibition (eg urinating in the kitchen sink at home); and short-term memory impairment. Mood inquiry revealed social withdrawal, irritability, and hypersomnia, but no other depressive symptoms (eg sadness, guilt, worthlessness). Psychotic symptoms included a seemingly delusional belief that his mother required a specialized ketogenic diet. The patient was noted to be emotional, amotivated, withdrawn, angry and defensive on assessment. Aside from alcohol use, there were no other substances reported. The preliminary diagnostic impression was an unspecified psychotic disorder vs a mood disorder with a psychotic component, and he was admitted to acute care psychiatry for further assessment and management.

In hospital, psychotic symptoms were unresponsive to trials of methrotrimeprazine, flupenthixol, loxapine, and paliperidone with notable intolerances including excessive drooling. There was no response to benzodiazepines nor 5 electroconvulsive treatments. Over the course of approximately two months in hospital, there was significant functional decline including purposeless repetitive behaviours, hyperphagia of simple carbohydrates, poor hygiene management, worsening confusion and cognition (Montreal Cognitive Assessment (MoCA) score 5/30), and signs of regression (eg, walking around the unit holding a teddy bear). He commented on experiencing visual hallucinations of his daughter; no other perceptual disturbances were noted. At approximately 3 months from the time of admission, he had become dependent for several activities of daily living including bathing, grooming and continence. His speech difficulties progressed to eventual mutism, and oral intake declined as dysphagia progressed. He walked purposelessly around the unit (up to 30 km/day). Neurological examination was unremarkable aside from mild coordination deficits (slightly slowed fine finger movements and toe tap bilaterally), and there was no fluctuation in symptoms to suggest delirium.

Concurrent with the progressive decline, an extensive work-up was conducted to establish diagnosis. It became clear early-on that the preliminary diagnosis of unspecified psychosis was unlikely given features such as late age of onset, lack of family history for a primary psychotic disorder, treatment non-response with several antipsychotic trials, as well as atypical features – most notably, the progressive cognitive and functional decline. In the context of increased disinhibition and regression, a rapidly progressive neurodegenerative disorder of unknown etiology was suspected.

In consultation with internal medicine, neurology, and geriatric psychiatry, multiple investigations were performed to assess for reversible causes of rapidly progressive dementia. Preliminary neuroimaging included a normal CT head and normal MRI brain aside from moderately dilated ventricles but no specific pattern of atrophy; there was no evidence of intracranial lesion, mass, infarction or hemorrhage. Routine blood work (CBC, electrolytes, extended electrolytes, renal and liver function) was unremarkable, with further tests demonstrating no evidence of systemic autoimmune or inflammatory (ANA, CRP, ESR), infectious (HIV, HBV, HCV, lyme, syphilis – RPR and VDRL, Whipple’s disease), vasculitis (negative vasculitis panel), toxic or heavy metal (lead, copper, iron and ceruloplasmin), endocrine (TSH, anti-TPO) or metabolic (plasma amino acid and urine amino and organic acid analysis) disease. Multiple causes of encephalitis were ruled out given no evidence of infection at any time, normal CT and MRI brain and negative CSF results for infectious, inflammatory, autoimmune (anti-NMDA), paraneoplastic (paraneoplastic antibodies) or neurodegenerative (CSF Tau protein, 14-3-3- protein, end-point quaking-induced conversion time was negative) causes. There were no features suggestive of an alcohol-related cause (eg Wernicke’s or hepatic encephalopathy), and liver enzymes, liver function tests (INR, PTT, albumin), and ammonia levels were within normal range. Neoplastic or paraneoplastic causes were unsupported by normal serum markers, SPEP, and CT chest/abdomen/pelvis showing no evidence of malignancy (there was note of very mild splenomegaly). Pertinent family history (medical or psychiatric) was bulbar ALS in the patient’s father who was diagnosed at the age of 54, and whose disease progressed to death within 18 months. This raised the possibility of a FTD-ALS genetic disorder in our patient.

A brain SPECT scan was ordered and demonstrated moderate decreased perfusion to the parietal lobes bilaterally, left greater than right, with some involvement of the posterior aspect of the left frontal lobe. The nuclear medicine radiologist concluded the distribution was most in keeping with Alzheimer’s disease and it was not felt to represent frontotemporal lobar degeneration (FTLD). However, given ongoing clinical suspicion for a rapidly progressive neurodegenerative cause, genetic testing was performed and identified a repeat expansion of GGGGCC in *C9orf72*, thereby confirming a diagnosis of FTD-ALS. The patient’s substitute decision maker was informed and goals of care were established given the progressive and ultimately fatal outcome of this disease. The genetics team played a central role in providing psychoeducation, counseling and support to the family.

The patient was transferred to a geriatric psychiatry inpatient unit for end-of-life care. Agitation and distress were treated symptomatically with 1:1 care, olanzapine and lorazepam as needed, and a modified diet for progressive dysphagia. Ultimately, he died from complications of aspiration pneumonia. His family consented to post-mortem autopsy and the final report confirmed a fronto-temporal lobar degeneration with transactive response DNA binding protein (TDP) pathology, international harmonized classification type C.

## Discussion

This is a case summary of a 42-year-old male with a rapidly progressive FTD-ALS caused by a pathogenic repeat expansion of GGGGCC in the *C9orf72* gene, which presented with psychosis and disorganized behavior leading to admission to psychiatry. This was on the background of typical symptoms of bvFTD such as disinhibition, apathy, carbohydrate craving and personality changes. Cognition and function rapidly declined over the course of several months. He died approximately two years after symptom onset. There were several salient features of this case that present learning opportunities to support more timely diagnosis of this relatively rare diagnosis.

### Diagnosis

Various elements of FTD-ALS presentations can lead to delay in diagnosis, including the overlapping yet heterogenetic neuropsychiatric clinical manifestations. Psychotic symptoms and other neuropsychiatric symptoms (eg apathy, disinhibition) are common presenting symptoms^12^ often misdiagnosed as a primary psychiatric disorder. In this case, although there were typical symptoms of bvFTD, the age of onset and presence of psychosis led to an initial diagnosis of an unspecified psychotic disorder. Psychotic symptoms, including delusions and/or hallucinations, are relatively more common in *C9orf72* positive cases of FTD-ALS than in *C9orf72* negative cases. Delusions (but not hallucinations) are also more common in FTD-ALS than in bvFTD without ALS^13^ As such, it was interesting that in this case (*c9orf72* positive FTD-ALS) the patient had visual hallucinations, as these are relatively rare in FTLD^14^ and in cases of psychosis amongst *C9orf72* carriers.^15^

In addition, other reports have described various psychiatric presentations of FTD-ALS, including mania, major depressive episodes with/without catatonic features, and obsessive-compulsive disorder. Of note, although bvFTD is commonly misdiagnosed as depression upon initial presentation, the symptoms leading to this diagnosis are often not specific to mood (eg guilt, worthlessness, crying), but more commonly apathy, reduced empathy, and social withdrawal.^16^ Careful clinical assessment can help to distinguish these features.

Patients with FTD-ALS *C9orf72* also have a higher rate of family psychiatric history^17^ which can lead to clinical bias and misdiagnosis.^16^ Furthermore, *C9orf72* gene expansions have also been identified as rare causes of other neurodegenerative phenotypes,^18^ including in 1% of Parkinson’s disease and Alzheimer’s disease,^3,4^ progressive supranuclear palsy, ataxic syndromes, corticobasal syndrome, Huntington disease-like syndrome, and Creutzfeldt-Jacob disease. Initial steps towards improving clinical care include awareness of the various potential clinical presentations.^11^

Ultimately, an atypical psychiatric presentation should lead to further investigations and consideration of involvement of other services. For example, some reports describe poor response and adverse reactions to antipsychotic medications^19,20^ which should trigger further investigations. Furthermore, family history is extremely important, and should include inquiries into early-onset dementia, FTD, ALS, Parkinsonism, and unexplained neuropsychiatric symptoms.

Clinical symptoms of dementia should be investigated with standardized testing and collateral. All atypical presentations should receive a minimum of a CT scan at baseline, and ideally an MRI to detect early signs of cortical and subcortical atrophy.^6^ The typical pattern of cognitive impairment seen in FTD-ALS includes deficits of social cognition and executive dysfunction and may include memory disturbances and parietal lobe deficits.^7,9^ Useful diagnostic criteria to aid in diagnosis include the revised Strong Criteria for diagnosis of frontotemporal dysfunction in ALS^21^ as well as the Neary^22^ or Hodges^23^ criteria for FTD-ALS.

### Genetic Testing

Technological advances in sequencing are allowing for genetic testing to play a useful role in the confirmation of inherited neurodegenerative disorders. Finding a genetic cause can be helpful as it can remove diagnostic uncertainty and inform affected families of their risk. It is also becoming increasingly useful to help qualify for precision treatment and to allow for opportunities to enrol into clinical trials.^24^

However, as *C9orf72* mutations are repeat expansions, they cannot always be accurately detected by the testing method employed for panels and whole exome sequencing. When investigating patients and choosing genetic testing for FTD-ALS, it is imperative to ensure that *C9orf72* is being sequenced; it is not currently included in all targeted genetic testing panels for early onset dementia and may need to be specifically ordered with a sample sent to an alternate laboratory for Sanger Sequencing.^24^ Whole genome sequencing in contrast has been shown to detect most repeat expansions for these conditions,^25,26^ but is not yet widely clinically available. This may evolve to be the test of choice in the future.

### Ethical Considerations

Despite the absence of disease modifying therapy, timely diagnosis of FTD-ALS has important implications for genetic family counselling and prognosis. Early diagnosis can also help to avoid further treatment trials and investigations, which carry various risks. As these illnesses can affect adults in the younger age (with age of onset identified from 27-83, and the disease duration is from 1-22 years), it can cause significant burden on patients and their families. Once a *C9orf72* pathogenic repeat has been identified as the cause of the FTD-ALS in the patient, genetic counselling is available to educate the family about the early signs and symptoms of the condition, phenotypic spectrum, inheritance pattern and the chance to inherit the condition. Family members may have the option of predictive genetic testing to determine if they have inherited the condition. Some individuals may use this information to make life and family planning decisions. For some, the option of pre-implantation genetic diagnosis may be available to avoid passing on the condition to future generations. Overall, diagnosis can help patients and families prepare for the devastating road ahead.

It is important to consider the ethical issues of genetic testing for adult-onset disorders and to obtain informed consent before ordering these tests. The process of consent differs, dependent upon whether the testing is being performed on a symptomatic patient to confirm a diagnosis or is predictive testing in an at-risk individual. When ordering testing on affected patients, they often have limited capacity to participate in informed decision making, and consent for genetic testing is often provided through the substitute decision maker. As these conditions are adult-onset and inherited in an autosomal dominant fashion, achieving a genetic diagnosis for early onset neurodegenerative conditions often has direct implications for family members.

Genetic testing in pre-symptomatic family members should only be undertaken after an extensive multi-session genetic counselling approach adapted from the protocol utilized in Huntington’s disease has been completed.^27,28^ The implications these conditions have for pre-symptomatic individuals including education, employment, insurance, and family planning, are daunting. Concern of how to communicate information about these conditions to other family members and especially their children, feeling frantic, or feeling of worry about symptom development, are all common reactions. For *C9orf72* patients in particular, the double threat of either developing dementia or ALS is particularly difficult and having the opportunity to explore their situation with genetic counsellors can be therapeutic.^29^

### Future Directions

Finally, an improvement into our understanding of these clinical manifestations and even possible “prodromes” in leading up to diagnosis will improve overall clinical care. The identification of a genetic cause of FTD-ALS has helped in understanding these illnesses^1,2^ and launched a significant amount of research on these topics.^6,30^ Indeed, there has been recent progress in developing targeted therapies for *C9orf72* related FTD-ALS. However, the development of these has been challenging, in that there may be both a toxic gain of function effect from the accumulation of expanded RNA foci and the accumulation of abnormal dipeptide repeat proteins (DPRs), as well as loss of function effect from a reduction in translation of C9orf72.^31^ Despite the challenges, antisense oligonucleotide therapeutics to bind and remove toxic RNA are being clinically developed.^32,33^ Small molecule approaches, such as repurposing Metformin to reduce toxic C9orf72 translation products, and new molecules such as TPN101 to reduce CNS inflammation are also in development.^34^ In addition, molecules have been studied to reduce the neurotoxic effects of DPRs by blocking their export from the nucleus.^35^ Loss of function effects have been less frequently studied, but new knockdown mouse models are now allowing researchers to further assess these effects.^36^ In this model, C9orf72 loss of function has been shown to be associated with the development of FTD-like symptoms,^36^ paving the way for deeper understanding of the effect of *C9orf72* expansions in FTD-ALS and ultimately help develop new therapeutic approaches. Most importantly, research is giving hope to families affected by this rare neurological condition, offering the potential of better treatments in the future and perhaps even a cure for this devastating condition.

## Conclusion

This paper described an interesting case of a 42-year-old male with psychiatric symptoms who was ultimately discovered to be a carrier of a *C9orf72* repeat expansion and diagnosed with FTD-ALS. *C9orf72* carriers may initially present with neuropsychiatric symptoms that appear to be a psychiatric disorder but are actually psychiatric symptoms in the context of a neurodegenerative disorder. Although this is often difficult to distinguish on initial presentation (eg whether mood related symptoms are in keeping with a mood or psychotic disorder vs an evolving neurodegenerative disorder), clinicians should consider neurodegenerative disorders in their differential diagnosis and diagnostic workup, especially when drug treatments for psychiatric disorders are failing, as occurred in this case. In addition, while FTD-ALS is rare, it is likely under diagnosed given the overlap between psychiatric and dementia/neurodegenerative phenotypes. This presentation highlights the need for genetic testing as part of this diagnostic workup, especially in younger, treatment resistant individuals and in the context of a positive family history of early onset degenerative neurological disorders.

Finally, this case report also highlights several key learning points related to making the diagnosis of FTD-ALS. Firstly, the importance of keeping a broad differential for presentations of atypical psychosis – in this case, with late age of onset, absence of family history, marked treatment resistance (to both antipsychotics and ECT), and significant functional decline beyond what would be expected in a primary psychotic disorder such as schizophrenia. Secondly, the important role of medical genetics and testing in psychiatric diagnoses, particularly in the context of the relevant family history. Thirdly, returning to the initial history in the context of diagnostic uncertainty, especially in the face of unexpected or non-confirmatory test results; in this case, although the neuroimaging findings were not consistent with FTD, the history led to further pursuance of genetic testing which ultimately confirmed the diagnosis. A diagnosis can prevent additional investigations, give the patient/family an explanation of why this disease has occurred, and offer an opportunity for patients and their families to receive supportive care through medical genetic counselling services.

## References

[1] Renton AE Majounie E Waite A , et al. A hexanucleotide repeat expansion in C9ORF72 is the cause of chromosome 9p21-linked ALS-FTD. Neuron. 2011;72(2):257-268. 21944779 10.1016/j.neuron.2011.09.010 PMC3200438

[2] DeJesus-Hernandez M Mackenzie IR Boeve BF , et al. Expanded GGGGCC hexanucleotide repeat in noncoding region of C9ORF72 causes chromosome 9p-linked FTD and ALS. Neuron. 2011;72(2):245-256. 21944778 10.1016/j.neuron.2011.09.011 PMC3202986

[3] Chiò A Borghero G Restagno G , et al. Clinical characteristics of patients with familial amyotrophic lateral sclerosis carrying the pathogenic GGGGCC hexanucleotide repeat expansion of C9ORF72. Brain. 2012;135(Pt 3):784-793. 22366794 10.1093/brain/awr366 PMC3286333

[4] Cruts M Gijselinck I Van Langenhove T van der Zee J Van Broeckhoven C . Current insights into the C9orf72 repeat expansion diseases of the FTLD/ALS spectrum. Trends Neurosci. 2013;36(8):450-459. 23746459 10.1016/j.tins.2013.04.010

[5] Lualdi M Shafique A Pedrini E , et al. C9ORF72 repeat expansion affects the proteome of primary skin fibroblasts in ALS. Int J Mol Sci. 2021;22(19):10385. 34638725 10.3390/ijms221910385 PMC8508815

[6] Ducharme S Bajestan S Dickerson BC Voon V . Psychiatric presentations of C9orf72 mutation: what are the diagnostic implications for clinicians? J Neuroparasitol. 2017;29(3):195-205. 10.1176/appi.neuropsych.16090168 28238272

[7] Rohrer JD Isaacs AM Mizielinska S , et al. C9orf72 expansions in frontotemporal dementia and amyotrophic lateral sclerosis. Lancet Neurol. 2015;14(3):291-301. 25638642 10.1016/S1474-4422(14)70233-9

[8] Rascovsky K Hodges JR Knopman D , et al. Sensitivity of revised diagnostic criteria for the behavioural variant of frontotemporal dementia. Brain. 2011;134(Pt 9):2456-2477. 21810890 10.1093/brain/awr179 PMC3170532

[9] Snowden JS Rollinson S Thompson JC , et al. Distinct clinical and pathological characteristics of frontotemporal dementia associated with C9ORF72 mutations. Brain. 2012;135(Pt 3):693-708. 22300873 10.1093/brain/awr355 PMC3286329

[10] Dobson-Stone C Hallupp M Bartley L , et al. C9ORF72 repeat expansion in clinical and neuropathologic frontotemporal dementia cohorts. Neurology. 2012;79(10):995-1001. 22875086 10.1212/WNL.0b013e3182684634 PMC3430710

[11] Ducharme S Price BH Larvie M Dougherty DD Dickerson BC . Clinical approach to the differential diagnosis between behavioral variant frontotemporal dementia and primary psychiatric disorders. Am J Psychiatry. 2015;172(9):827-837. 26324301 10.1176/appi.ajp.2015.14101248

[12] Snowden JS Harris J Richardson A , et al. Frontotemporal dementia with amyotrophic lateral sclerosis: A clinical comparison of patients with and without repeat expansions in C9orf72. Amyotroph Lateral Scler Frontotemporal Degener. 2013;14(3):172-176. 23421625 10.3109/21678421.2013.765485

[13] Long Z Irish M Foxe D Hodges JR Piguet O Burrell JR . Heterogeneity of behavioural and language deficits in FTD-MND. J Neurol. 2021;268(8):2876-2889. 33609157 10.1007/s00415-021-10451-7

[14] Naasan G Shdo SM Rodriguez EM , et al. Psychosis in neurodegenerative disease: Differential patterns of hallucination and delusion symptoms. Brain. 2021;144(3):999-1012. 33501939 10.1093/brain/awaa413 PMC8041322

[15] Devenney EM Landin-Romero R Irish M , et al. The neural correlates and clinical characteristics of psychosis in the frontotemporal dementia continuum and the C9orf72 expansion. Neuroimage Clin. 2016;13:439-445. 28116236 10.1016/j.nicl.2016.11.028 PMC5233794

[16] Woolley JD Khan BK Murthy NK Miller BL Rankin KP . The diagnostic challenge of psychiatric symptoms in neurodegenerative disease: rates of and risk factors for prior psychiatric diagnosis in patients with early neurodegenerative disease. J Clin Psychiatry. 2011;72(2):126-133. 21382304 10.4088/JCP.10m06382oli PMC3076589

[17] Devenney E Hornberger M Irish M , et al. Frontotemporal dementia associated with the C9ORF72 mutation: A unique clinical profile. JAMA Neurol. 2014;71(3):331-339. 24445580 10.1001/jamaneurol.2013.6002

[18] Woollacott IOC Mead S . The C9ORF72 expansion mutation: Gene structure, phenotypic and diagnostic issues. Acta Neuropathol. 2014;127(3):319-332. 24515836 10.1007/s00401-014-1253-7

[19] Landqvist Waldö M Gustafson L Nilsson K , et al. Frontotemporal dementia with a C9ORF72 expansion in a Swedish family: Clinical and neuropathological characteristics. Am J Neurodegener Dis. 2013;2(4):276-286. 24319645 PMC3852567

[20] Proudfoot M Gutowski NJ Edbauer D , et al. Early dipeptide repeat pathology in a frontotemporal dementia kindred with C9ORF72 mutation and intellectual disability. Acta Neuropathol. 2014;127(3):451-458. 24445903 10.1007/s00401-014-1245-7

[21] Strong MJ Abrahams S Goldstein LH , et al. Amyotrophic lateral sclerosis - frontotemporal spectrum disorder (ALS-FTSD): revised diagnostic criteria. Amyotroph Lateral Scler Frontotemporal Degener. 2017;18(3-4):153-174. 28054827 10.1080/21678421.2016.1267768 PMC7409990

[22] Neary D Snowden JS Gustafson L , et al. Frontotemporal lobar degeneration: A consensus on clinical diagnostic criteria. Neurology. 1998;51(6):1546-1554. 9855500 10.1212/wnl.51.6.1546

[23] Hodges JR Miller B . The classification, genetics and neuropathology of frontotemporal dementia. Introduction to the special topic papers: Part I. Neurocase. 2001;7(1):31-35. 11239074 10.1093/neucas/7.1.31

[24] Huq AJ Sexton A Lacaze P , et al. Genetic testing in dementia-A medical genetics perspective. Int J Geriatr Psychiatry. 2021;36(8):1158-1170. 33779003 10.1002/gps.5535

[25] Huq AJ Thompson B Bennett MF , et al. Clinical impact of whole-genome sequencing in patients with early-onset dementia. J Neurol Neurosurg Psychiatry 2022;2021:328146. 10.1136/jnnp-2021-328146 35906014

[26] Ibañez K Polke J Hagelstrom RT , et al. Whole genome sequencing for the diagnosis of neurological repeat expansion disorders in the UK: A retrospective diagnostic accuracy and prospective clinical validation study. Lancet Neurol. 2022;21(3):234-245. 35182509 10.1016/S1474-4422(21)00462-2 PMC8850201

[27] Roberts JS Patterson AK Uhlmann WR . Genetic testing for neurodegenerative diseases: Ethical and health communication challenges. Neurobiol Dis. 2020;141:104871. 32302673 10.1016/j.nbd.2020.104871 PMC7311284

[28] Goldman JS Hahn SE Catania JW , et al. Genetic counseling and testing for alzheimer disease: Joint practice guidelines of the American college of medical genetics and the national society of genetic counselors. Genet Med. 2011;13(6):597-605. 21577118 10.1097/GIM.0b013e31821d69b8 PMC3326653

[29] Dratch L Owczarzak J Mu W , et al. The lived experience of reconstructing identity in response to genetic risk of frontotemporal degeneration and amyotrophic lateral sclerosis. J Genet Couns. 2023. 10.1002/jgc4.1749 PMC10776796 37424394

[30] Balendra R Isaacs AM . C9orf72-mediated ALS and FTD: multiple pathways to disease. Nat Rev Neurol. 2018;14(9):544-558. 30120348 10.1038/s41582-018-0047-2 PMC6417666

[31] Dane TL Gill AL Vieira FG Denton KR . Reduced C9orf72 expression exacerbates polyGR toxicity in patient iPSC-derived motor neurons and a Type I protein arginine methyltransferase inhibitor reduces that toxicity. Front Cell Neurosci. 2023:17. https://www.frontiersin.org/articles/10.3389/fncel.2023.1134090 Accessed July 21, 2023. 10.3389/fncel.2023.1134090 PMC10149854 37138766

[32] Tran H Moazami MP Yang H , et al. Suppression of mutant C9orf72 expression by a potent mixed backbone antisense oligonucleotide. Nat Med. 2022;28(1):117-124. 34949835 10.1038/s41591-021-01557-6 PMC8861976

[33] Liu Y Dodart JC Tran H , et al. Variant-selective stereopure oligonucleotides protect against pathologies associated with C9orf72-repeat expansion in preclinical models. Nat Commun. 2021;12(1):847. 33558503 10.1038/s41467-021-21112-8 PMC7870851

[34] Zampatti S Peconi C Campopiano R Gambardella S Caltagirone C Giardina E C9orf72-Related neurodegenerative diseases: from clinical diagnosis to therapeutic strategies. Frontiers in Aging Neuroscience 2022;14. https://www.frontiersin.org/articles/10.3389/fnagi.2022.907122 Accessed July 21, 2023. 10.3389/fnagi.2022.907122 PMC9226392 35754952

[35] Castelli LM Lin Y-H Sanchez-Martinez A , et al. A cell-penetrant peptide blocking *C9ORF72*-repeat RNA nuclear export reduces the neurotoxic effects of dipeptide repeat proteins. Sci Transl Med. 2023;15(685):eabo3823. 36857431 10.1126/scitranslmed.abo3823

[36] Lopez-Herdoiza MB Bauché S Wilmet B , et al. C9ORF72 knockdown triggers FTD-like symptoms and cell pathology in mice. Front Cell Neurosci. 2023;17:1155929. 37138765 10.3389/fncel.2023.1155929 PMC10149765

